# Interaction of Long Time Pulses of an Nd^3+^:YAG Laser Beam with the Heusler AlloyNi_45_Co_5_Mn_35.5_In_14.5_

**DOI:** 10.3390/ma14227016

**Published:** 2021-11-19

**Authors:** Patryk Ciupak, Artur Barłowski, Piotr Sagan, Tadeusz Jasiński, Marian Kuzma

**Affiliations:** 1 Department of Physics and Medical Engineering, The Faculty of Mathematics and Applied Physics, University of Technology, al. Powstancow Warszawy 6, 35-959 Rzeszow, Poland; patryk.ciupak@gmail.com (P.C.); barlowskiartur@gmail.com (A.B.); 2 The Faculty of Mathematics, Physics, and Computer Science, Maria Curie-Sklodowska University Lublin, Plac Marii Curie-Skłodowskiej 5, 20-400 Lublin, Poland; psagan@poczta.umcs.lublin.pl; 3 Technical College, East European State University in Przemysl, Ksiazat Lubomirskich 6, 37-700 Przemysl, Poland; kuzma@univ.rzeszow.pl

**Keywords:** Ni-Mn-In Heusler alloy, laser processing, single-shot laser damage, hole drilling

## Abstract

In this paper, the laser processing of the surface of bulk and layered samples (of thickness 75 nm) of Ni_45_Co_5_Mn_35.5_In_14.5_ alloy (NC5MI) was investigated using microsecond laser pulses. A Q-switched pulsed Nd^3+^:YAG laser, operating in the 1st harmonic (which had a wavelength of 1064 nm) with a pulse duration of 250 µs, was used. NC5MI is a metal resistant to thermal laser processing because its reflection coefficient is close to unity for long wavelengths. The aim of this paper was to learn the forms of laser processing (heating, microprocessing, ablation) for which the above-specified type of laser is useful. The samples were irradiated with various fluences in the interval of 5–32 J·cm^−2^. The effect of the laser interaction with the surface was explored by SEM microscopy. The threshold fluences for the bulk sample were determined as: the visible damage threshold (F_th_^d^ = 2 ± 0.2 J·cm^−2^), the melting threshold (F_th_^m^ = 10 ± 0.5 J·cm^−2^), and the deep melting threshold (F_th_^dm^ = 32 J·cm^−2^). Unexpectedly, these values wereincreased for the layer sample due to its silicon substrate. We have concluded that this type of laser radiation is advantageous for the annealing and melting of, or drilling holes in, the alloy, but disadvantageousto the ablation of the alloy.

## 1. Introduction

Heusler alloys of the X-Y-Z type (X = Ni; Y = Mn, Co, Fe; Z = Ga, Sb, Sn, In) have many unique properties (such as the shape memory effect, high magnetoresistance, the magnetocaloric effect, etc.). These propertiesare attributed to a martensitic phase transition (MPT). MPT depends on the composition, stoichiometry, temperature, magnetic field, and thermal history of the sample [1,2,3]. They occur when the first order transition from the austenite to the martensite phase is at a temperature which coincides with the Curie temperature of the alloy [1]. Both temperatures depend strongly on composition [1,2]. Moreover, the crystallographic phase of the austenite phase should be cubic of the L2_1_ type. Using the conventional technique, this is done by long-time homogenization at 1173 K for 24 h and then quenching in water. Any subsequent annealing of the material can lead to damage of this structure and a disappearance of the material features listed above.

In order to manufacture devices (actuators, magnetic refrigerators, or hard discs) based on the MC5Ni Heusler alloy, it is necessary to manufacture this material in different forms. For example, in thin layers (for spintronic devices), thick layers (e.g., for actuators), or as printed components (for refrigerators). Such structures can certainly be obtained with the application of laser treatment. Picosecond lasers are applied to produce thin layers with the use of the pulsed laser deposition method (PLD). However, lasers with a longer pulse length (of the order of nanoseconds) must be used for heating thick layers to harden themto develop a martensitic phase. Moreover, long pulse lasers are useful for melting powder grains when the Direct Energy Deposition technology (DED) is used.

Therefore, the thermal laser processing of such materials—which is highly desirable for the innovative manufacturing of smart devices using pulsed laser deposition of thin layers or 3D printing—should be carefully experimentally tested before application.

The NC5MI alloy is a hard but very brittle material, which is the reason why it is resistant to the effects of laser irradiation, especially when the wavelength of the laser is increased. The purpose of this paper is to examine the effects of the action of a neodymium laser beam, of moderate power (up to 1 J per pulse), and long pulse time (250 microseconds) on the surfaces of two samples: a bulk sample and a sample in the form of a thick layer deposited on Si. The energy thresholds are determined for surface melting, deep melting, and ablation of the material.

The interaction of a laser beam with a metal surface causes unique changes in the material which cannot be achieved by conventional methods of surface processing (by thermal or chemical methods). Therefore, the laser treatment of metals has numerous applications in the metallurgical industry. The effect of the interaction of laser light with a metal depends on a wide range of parameters which can be divided into two sets:

(A) Material parameters, such as the coefficient of reflection, density, thermal and temperature conductivity, or temperature;
(B) Laser processing parameters, such as the wavelength, duration and shape of the laser pulse, its fluence, or the diameter of the laser spot.

Changes in the laser parameters lead to various physical mechanisms which modify the properties of the material irradiated. At the first stage, these mechanisms are divided into those which are thermal (for long-time pulses in the range of microseconds or nanoseconds) and those which are non-thermal (for femtosecond durations of pulses). One can determine whetherthe process is thermal if the laser-induced excitation rates are comparable to the thermalization time. In this case, we deal with the heating and cooling of the material in a small space, comparable with the diameter of the spot. During the heating, the material reaches a very high temperature in a short time and, therefore, the temperature gradients for heating and cooling are extremely high, reaching values as high as 10^9^ K/s for nanosecond pulses. However, the maximal temperature of the material achieved during laser heating depends on the energy density of the spot. The effects of irradiation on the surface can be various, depending on two fundamental parameters: the pulse duration and its energy density. For small fluences, below the threshold of melting, the near-surface layer may change its crystal structure (hardening may occur) or the segregation of impurities (doping) or sintering of complex materials may take place. The same processes occur in the molten substrate layer when the energy of the laser pulse is higher. After the melting, additional effects are important for future applications; for example, the removal of the material from the molten area which leads to such applications as the patterning of the surface, for cutting of thin sheets, or for drilling holes. An increase in the fluence results in the evaporation of the material [4]. This effect is utilized for the deposition of the functional layers of a very large group of materials, involving metals, semiconductors, oxide insulators, polymers [5], and such complex materials as high-T_c_ superconductors [6,7].

In this paper, we study the effects of the interaction of an Nd^3+^:YAG laser beam of moderate energy density with an Ni-Mn based Heusler alloy. We used the long-time pulses of the laser beam. We determined the kind of laser-induced damage to the sample surface, depending on the incident fluence of the laser beam. The results were compared with the interactions of the laser beam with a thin layer of the NC5MI deposited on silicon.

## 2. Experimental Section

The parameters of the Nd^3+^:YAG laser beam were as follows: wavelength 1060 nm (the first harmonic), pulse duration 250 μs, diameter of the laser beam 6 mm, maximal energy in the pulse 1 J. 

The bulk samples of Ni_45_Co_5_Mn_35.5_In_14.5_ were created by Maziarz [8] through induction melting in an argon atmosphere, using pure (9.99) elements. The layers of NC5MI were obtained by Wisz [9] using the PLD method on Si single crystal wafers with a (100) surface orientation.

**Figure 1:**
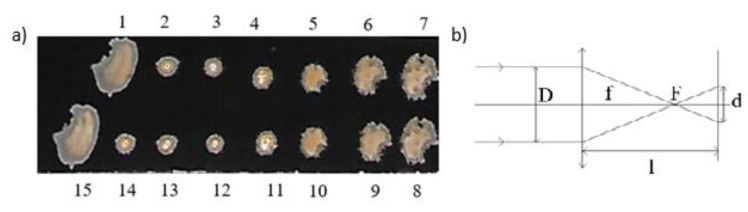
Spots of the focused laser beam on black photocopy paper (3× magnification) and numeration of the spots (**a**). The scheme for focusing the laser beam (**b**). The symbols in Figure (**b**) denotes: D—the laser beam diameter, F, f—the focal point and the focal length respectively, d—the spot diameter.

The samples were mounted on an XY holder. Using a 10D lens, the laser beam was focused on the surface of the sample, and various diameters of the beam spot were obtained by varying the distance l between the lens and the target (Figure 1b).

The traces of the interaction of the laser beam with the surface of the sample (the spots) were compared with the spots produced on black photocopy paper. From these, the diameter of the spots after focusing were determined. A set of the spots on photocopy paper at different lens-target distances is presented in Figure 1a. There are two sets of the spots obtained for the same distances l, which are numbered as 1–7 in the upper row, and as 8–15, in the bottom row, respectively. The similarity of these sets demonstrates the very high stability of the laser beam. The shape of the spots is elliptical (particularly for small focusing); thus, the diameter d of the spots was calculated as an average value of the two diameters D_1_ and D_2_ of the ellipse.

The shape and morphology of the spots presented in Figure 1 were used as a test for the laser energy calibration for essential experiments within the irradiation of the samples.The fluences of the laser beam were calculated for different diameters d of the spots by dividing the laser pulse energy E by the spot area S:

ε = E/S.(1)

The energy of the laser pulse was 0.77 J (at the discriminator “4” on the laser control system). These fluences have been collected in Table 1.

**Figure 2:**
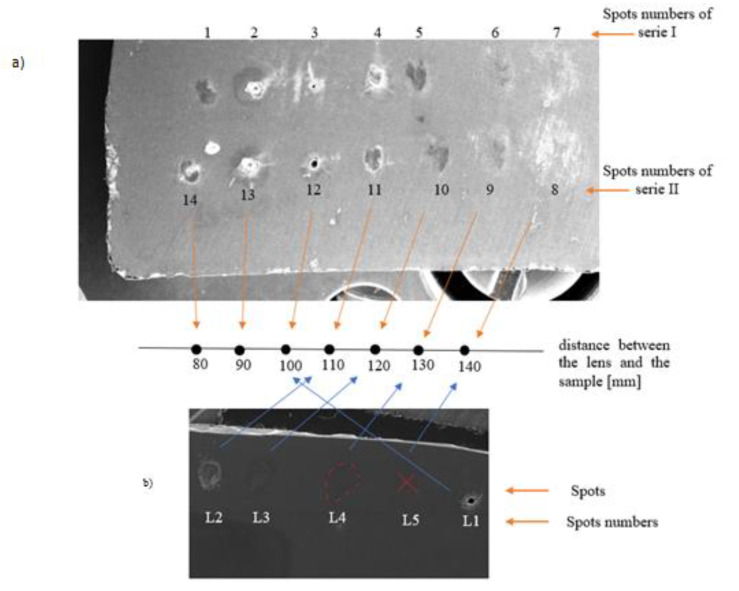
Laser beam spots (**a**) on the bulk sample of NC5MI; (**b**) on the layer of NC5MI deposited on Si.

Two series of the spots obtained on a bulk sample of NC5MI at different target–lens distances are presented in Figure 2a. In Figure 2b, the spots were obtained on a layered sample of NC5MI deposited on Si.

## 3. Results and Discussion

### 3.1. Bulk Sample

**Figure 3:**
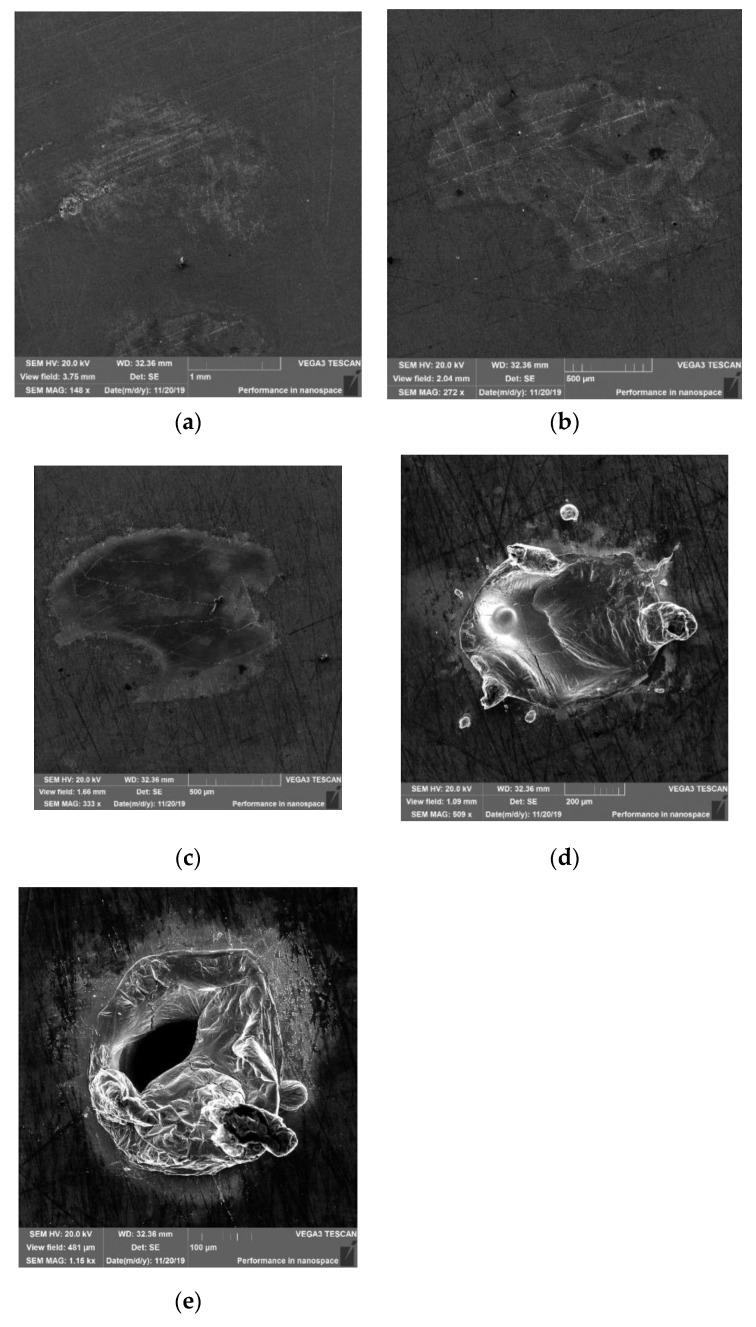
SEM micrographs of the spots on bulk samples for fluencies ε = 5.43; 6.97; 13.0; 24.5; 32.0 J·cm^−2^: (**a**) spot No. 7, ε = 5.43 J·cm^−2^; (**b**) spot No. 6, ε = 6.97 J·cm^−2^; (**c**) spot No. 5, ε = 13.0 J·cm^−2^; (**d**) spot No. 4, ε = 24.5 J·cm^−2^; (**e**) spot No. 3, ε = 32.0 J·cm^−2^.

The photographs of selected spots obtained on the bulk surface, taken using a scanning electron microscope (SEM) (Tescan Vega3SBH, Tescan Orsay Holding, Brno, Czech Republic) are shown in Figure 3.

A slight change in the texture of the bulk sample surface is observed at a low energy density (l = 14cm, ε = 5.43 J·cm^−2^) interaction of the laser beam with the surface (Figure 3a). However, after increasing the energy by up to 6.97 J·cm^−2^, small, melted pieces are visible within the area of the beam interaction with the surface (Figure 3b). Partial melting of the sample surface, or even its vaporization, occurred at an energy equal to 13.0 J·cm^−2^ (Figure 3c). The area of such vaporization was approximately 0.8 mm^2^. Further energy increases, up to 24.5 J·cm^−2^, resulted in considerable melting of the material, with a spout (Figure 3d), without a significant increase in the melted area, when compared to the energy 13.0 J·cm^−2^ (see spots 4 and 5 in Figure 2a, compared to the micrographs d, c in Figure 3). A significant change in the impact spot morphology can be noticed following another energy increase of up to 32 J·cm^−2^ (micrograph 3e). At such an energy (and when the sample is placed in the focus plane), the spot diameter changed significantly to 0.30 mm. This corresponds to the energy at which material is being melted and thrown outside in the form of an effluence.

**Figure 4:**
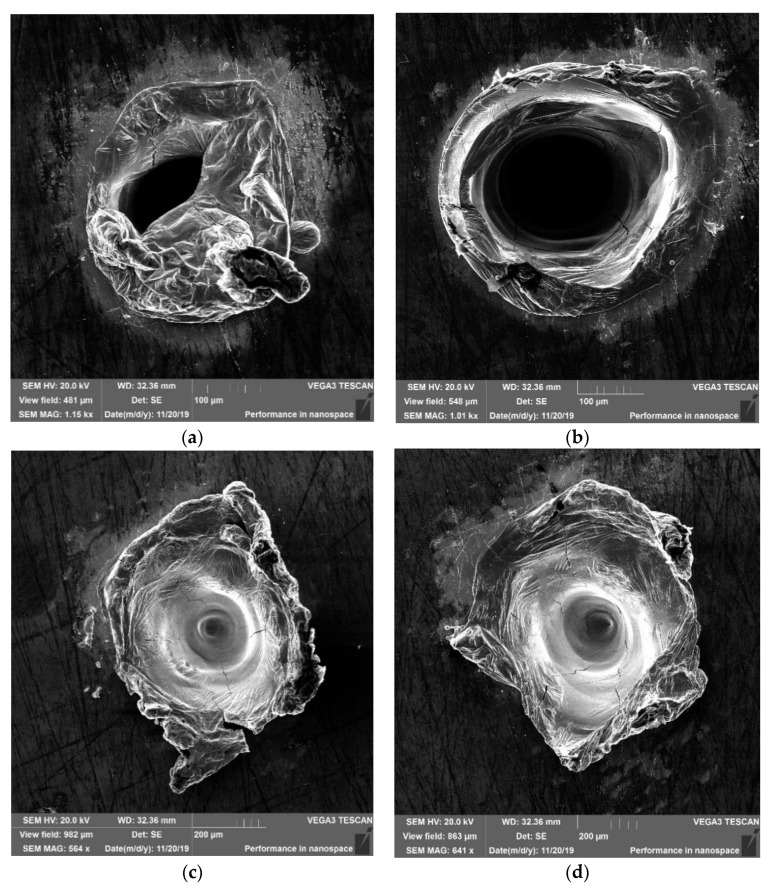
SEM micrographs of the spots on the bulk sample in series I (left) and series II (right): (**a**,**b**)—spots No. 3 and 12, ε = 32.0 J·cm^−2^; (**c**,**d**)—spots No. 2 and 13, ε = 27.8 J·cm^−2^.

In Figure 4, we have compared the spots obtained at the same fluence in two series of irradiations. At these selected fluences (32.0 J·cm^−2^ and 27.8 J·cm^−2^), the interaction of the laser beam with the target results in the deep melting of the surface, and the details of this damage, are easily observed in a visual comparison. The spots that did not melt, obtained at a lower fluence, may be compared in respect of their diameter only, which can be seen in Figure 2a (spots 5–10, 6–9, 7–8). Taking into account the size of the spots in both series (Figure 2) and the morphology of the spots in the case of deep melting (Figure 4), it can be concluded that consistent repetition of the effects of the interaction of a single laser pulse with the target occurs in both series, particularly at lower fluences, as shown in Figure 4c,d.

**Figure 5:**
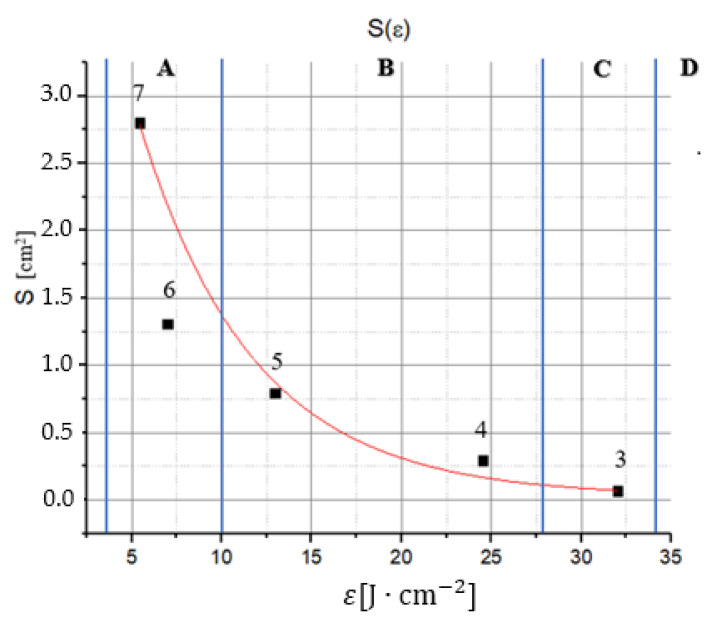
Dependence of the spot size on the fluence.

Figure 5 shows a diagram of the dependence of the laser beam impact spot size on the density of the energy applied for a bulk sample (Figure 2a). In the diagram, four areas of the characteristic impact, A, B, C, and D, can be distinguished. Below 2 J·cm^−2^, no signs of melting are observed, as only warming of the material occurs. Melting of the surface without its vaporization is observed (spots 6, 7—area A) at the energy levels 2–10 J·cm^−2^.Melting accompanied by surface vaporization of the material (traces 5, 4—area B) is observed within the energy range 10–28 J·cm^−2^. Deep melting, with effluence (trace 3—area C), is observed at the energy range 28–34 J·cm^−2^. Moreover, in the case of spot 12, having the same energy, deep melting occurs, but without effluence (Figure 4b). This results from the fact that there is a limit to the emergence of hollows and there are none within the energy range 32 ± 1 J·cm^−2^.

Area D is the energy range for making precise craters. However, the crater diameter does not change, and only its depth becomes greater within this range.

From the SEM micrographs presented in Figure 3 and from the diagram in Figure 5, we have estimated the single pulse laser-induced threshold fluences as the borders of the areas of the characteristic damage (bold vertical lines in the diagram in Figure 5). Thus, we have obtained the visible damage threshold (F_th_^d^ = 2 ± 0.2 J·cm^−2^), the melting threshold (F_th_^m^ = 10 ± 0.5 J·cm^−2^), and the deep melting threshold (F_th_^dm^ = 32 J·cm^−2^).

For comparison, we have calculated the vaporization threshold F_th_^v^, using the following Equation [10]:

F_th_^v^ = ρL_v_ α^1/2^ τ^1/2^ (2)

where ρ is the sample density, τ is the laser pulse width, L_v_ is the latent heat of evaporation, andthe thermal diffusivity α is

α = K/ρC_p_ (3)

where K is thermal conductivity and C_p_ is specific heat.

For the values taken from Table 2, for the Ni-Mn-In alloy, ρ = 7.7 g/cm^3^, L_v_ = 6200 J/g (the averaged value of those for Fe and Ni) and τ = 250 µs, we have obtained: F_th_^v^ = 122 J·cm^−2^. It is far above our experimental fluences, which means that the pure evaporation effect is not available in this laser configuration (wavelength, pulse duration, and maximum energy).

### 3.2. Thin Layer Sample

The spots of the laser beam interaction with a thin NC5MI layer, deposited on a monocrystalline silicone using the PLD method, are presented in Figure 2b and marked as L1—L5. This numeration does not correspond to the marking of the spots for the bulk sample in Figure 2a. Nevertheless, the spots are assigned to the lens distance from the sample at the relevant energy density.

**Figure 6:**
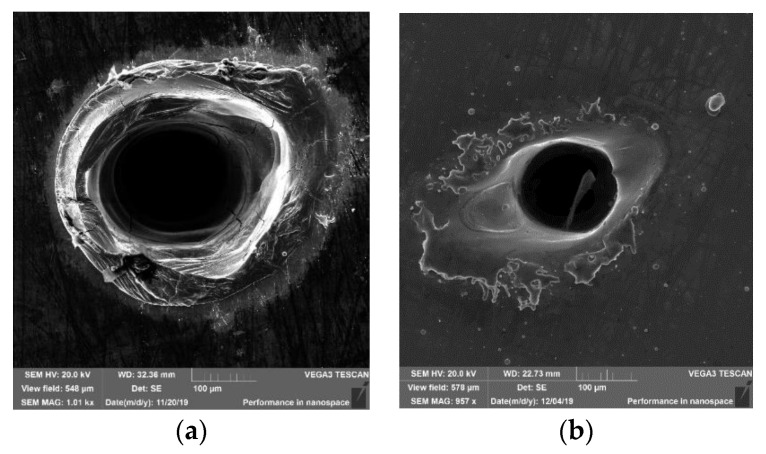
Craters obtained in (**a**) the bulk sample and in (**b**) the layer after maximal focusing of the laser beam.

An analysis of Figure 2b reveals that the spots on the layer are significantly less ‘clear’ than those in the bulk sample. For low energy densities, no visible changes in the layer morphology are found; e.g., for energy ε = 5.43 J·cm^−2^, the spot (L5) is invisible, while, in the bulk sample, such an energy density causes a noticeable impact effect visible as a spot with a wide diameter (see trace 8 or 7 in Figure 2a). However, for the large energy density, an effect identical to that on the bulk sample can be observed. Spots L1 on the layer and 12 on the bulk sample are almost identical (the distance of the samples from the lens was 10 cm). Those spots are deep craters, with a diameter of 0.230 ± 0.003 mm and 0.166 ± 0.003 mm for bulk and layered samples, respectively (see Figure 6).

A summary of the impact effects for the bulk sample and the layer sample have been presented in Table 3.

The impact type is identical for high energy density (ε = 32 J·cm^−2^), namely for deep craters creation. As the energy density is decreased, an increase in the differences between both the morphology and the diameters of the spots is observed. For energy ε = 13 J·cm^−2^, the spot on the layer is almost twice smaller, compared to that on the solid sample, whereas, for energy ε = 5.43 J·cm^−2^, no trace on the layer is observed, while it is clearly visibly on the solid sample.

The differences observed in the spot morphology can be explained by the poorer absorption of laser radiation by the surface of the layer. The surface of the solid sample was metallic, sanded but not polished, and its coarseness was average; consequently, the laser beam reflection coefficient was not high, which made the absorption easier. In contrast, the layer sample examined was mirror-like, meaning that its reflection coefficient was incredibly high—consequently, the absorption of radiation by the layer was made difficult. It is only at high energy densities (where non-linear effects of the radiation absorption occur) that the reflection coefficient does not play an important part in the absorption—thus, such an effect was observed for the highest energy density at 32 J·cm^−2^.

**Figure 7:**
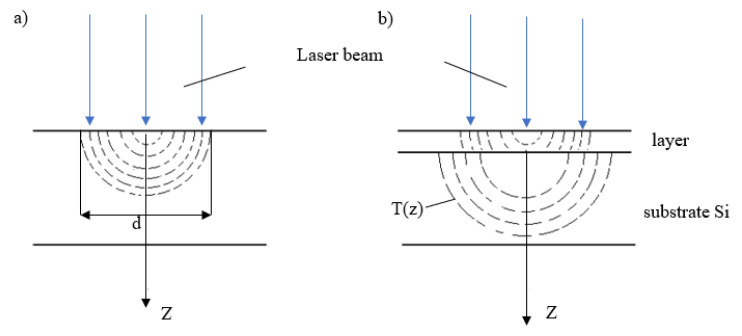
Thermal conductivity at laser heating in (**a**) the bulk sample and (**b**) the layer sample.

Another factor causing the differences in the spot morphology is the thermal conductivity coefficient in the solid sample. If thermal conductivity is high, the energy absorbed by the sample spreads hemispherically (Figure 7a) in all directions, thus facilitating the absorption of radiation. The process in which the thermal energy spreads when the examined material is either a layer or a substrate has been presented in Figure 7b.

The layer–substrate boundary causes a significant change in both the thermal conductivity coefficient and the reflection coefficient. A change in the reflection coefficient is of no relevance, as laser radiation penetrates only a shallow distance into the examined layer due to its being a metallic sample (light cannot penetrate deeply into metal). However, the thermal conductivity coefficient changes, as the substrate is silicon, i.e., a semiconductor in which the electron density is far smaller than the electron density in a metal. The electron density is approximately 10^22^·c·m^−3^ in a Heusler alloy, whereas it is 10^16^·c·m^−3^ in silicon. According to Franz’s law, thermal conductivity is proportional to electron density—consequently, thermal conductivity in silicon should be several orders lower than in a metal. In fact, phonon thermal conductivity exceeds electron thermal conductivity, ensuring very high thermal conductivity K in silicon (see Table 2), which is one order higher when compared to stainless steel. Zheng et al. [14] have noticed that the thermal constants (thermal conductivity and thermal diffusivity) of the Ni-Mn-In alloys differs significantly from such metals as Ag, Ni, or steel (Fe)—see Table 2. On the contrary, these constants are comparable to those for stainless steels. Hence, it is assumed that the thermal energy in the layer reaches the substrate at the same time as in the solid sample, but there is a faster movement of the thermal energy delivered in silicon. Hence, the energy absorbed on the layer surface warms this layer to the melting temperature more slowly than in the solid sample, and the surface may not even reach the melting temperature, while the pulse lasts at low energy values. Consequently, the spots on the layer should be linked to the material vaporization mode, expressed by the depth equal to the layer thickness. Nevertheless, this remains contrary to what is observed experimentally—only material overheating is observed. Only spot No. 2 on the layer can be interpreted as the vaporization of the layer without any damage to the substrate, which is silicon here. 

From the SEM image in Figure 2b and from Table 3, one can estimate the single shot melting threshold on F_th_^m^ (layer) = 12 ± 0.5 J·cm^−2^. This value is greater than that of the bulk sample (10 J·cm^−2^), which results from the influence of the Si substrate on the heating process. This result is in contrast with the studies by Matthias et al. [15], who have showed a linear dependence (decrease) of the ablation and the damage thresholds on film thickness for Au and Ni films. However, this dependence has been observed only for a film thickness smaller than the thermal diffusion length.

L_th_ = (2ατ)^1/2^ (4)

The length calculated for our material was L_th_ = 110 nm, whereas the thickness of the layered sample was 75 nm [9]. Therefore, we are in the position to exclude the dependence of the layer thickness on an increase in the threshold fluence.

## 4. Conclusions

In this paper, we have shown that long pulses of an Nd^3+^:YAG laser can have a significant effect on the annealing and melting of the surface of an Ni_45,5_Co_5_Mn_35.5_In_14.5_ alloy at moderate fluences of the laser beam (5–32 J·cm^−2^). For the bulk sample, we have determined the single-shot damage thresholds: the visible damage threshold (F_th_^d^ = 2 ± 0.2 J·cm^−2^), the melting threshold (F_th_^m^ = 10 ± 0.5 J·cm^−2^), and the deep melting threshold (F_th_^dm^ = 32 J·cm^−2^). We have shown that, for the layered sample, these values are increased due to its silicon substrate, which has extraordinary thermal properties. At higher fluences, well-formed hole drilling is observed. The hole drilling in metals using long-pulse Nd:YAG lasers has been studiedexperimentally and modeled mathematically since the very first applications of these lasers in industry. Nevertheless, this type of research is current for new materials and new applications [16,17,18]. Therefore, such lasers may be used as flexible tools for drilling holes in bulk samples of the Heusler alloys, which may be applied in magnetocaloric refrigeration for forming heat exchange channels, as this material shows excellent magnetocaloric properties [19,20,21]. Moreover, it seems that greater pulse lengths may be more useful in the field of powder metallurgy, where such pulses would not interfere with the stoichiometry of the multicomponent alloys (it should be noted that the Heusler alloys studied here are very sensitive to their composition [22]). This property should be considered when developing the 3d printing of such materials. Recently, numerous publications have appeared on the subject, including phase transformations, after laser printing, in Heusler alloys (Ni-Mn-Ga, Ni-Mn-Sn) [23,24,25,26]. The overall conclusion is that the additional classic annealing should be used in order to restore the initial microstructural features of the alloys [27,28].

## Figures and Tables

**Table 1:** Averaged diameter d of the spots in Figure 1 and the incident fluences ε (the diameters and fluences of the spots in series II are the same as for the spots in series I).

Series I Spot No	Series II Spot No	l_1_ [cm]	D_1_ [mm]	D_2_ [mm]	d [mm]	S [mm^2^]	ε [J·cm^−2^]
1	15			unfocused beam			
2	13, 14	9	2	1.75	1.875	2.76	27.8
3	12	10	2	1.55	1.75	2.40	32.0
4	11	11	2	2	2	3.14	24.5
5	10	12	2.5	3	2.75	5.93	13.0
6	9	13	3.5	4	3.75	11.04	6.97
7	8	14	5	3.7	4.25	14.18	5.43

**Table 2:** Physical constants of the materials considered (partially according to Table 1 of [10]).

Elements or Alloys	ρ [g·cm^−3^]	L_v_ [J·g^−1^]	L_f_ [J·g^−1^]	C_p_ [J·K^−1^·kg^−1^]	K [Wm^−1^·γ^−1^]	A [cm^2^·s^−1^]	U_coh_ [kJ/mol] [11]
Ag	10.50	2390	103	237	429	1.72	348.75
Ni	8.90	6378	292	444	91	0.23	497.76
Fe	7.87	6095	272	444	80	0.23	528.81
Stainless steel	7.7–8.0			482	10–30	0.04	445.35
Ni_50_Mn_50−x_In_x_ X = 14–14.7	7.8			[12,13]	[14]		
-austenite (A)				425–450	11.5–13.0	0.03	
-martensite (M)				450	7.0–8.5	0.025	
-near temp. of M-A transition				550	5.5–13		
Si	2.33	1370	142	700	148	0.88	502.00

L_v_—latent heat of evaporation; L_f_—latent heat of fusion; C_p_—specific heat; K—thermal conductivity; α = K/ρC_p_—thermal diffusivity; U_coh_—cohesive energy.

**Table 3:** Description of the spots in Figure 2.

	Bulk Sample				Layered Sample			
ε [J·cm^−2^]	Spot No.	d [mm]	Spot Morphology	Type of Interaction	Spot No.	d [mm]	Spot Morphology	Type of Interaction
32.0	3	0.230	deep crater	hole drilling	L1	0.166	deep craters	hole drilling
24.5	4	0.610	visible: effusion of material	melting, with effusion	L2	0.583	melted layer of material	surface evaporation
13.0	5	1.005	visible: some spots are clearly melted, with evaporation	melting, with surface evaporation	L3	0.657	less clear and smaller spots, compared to the bulk sample	melting, without evaporation
6.97	6	1.290	partially melted	melting, without evaporation	L4	835.75	slightly changed colour	slight melting, annealing
5.43	7	1.890	colour changing	no melting	L5	-	no spots	annealing

## Data Availability

The data presented in this study are available on request from the corresponding author.
